# Toward Objective Assessment of Positive Affect: EEG and HRV Indices Distinguishing High and Low Arousal Positive Affect

**DOI:** 10.3390/s26020521

**Published:** 2026-01-13

**Authors:** Yuri Nakagawa, Tipporn Laohakangvalvit, Toshitaka Matsubara, Keiko Tagai, Midori Sugaya

**Affiliations:** 1 Functional Control Systems, Shibaura Institute of Technology Graduated School, 3-7-5 Toyosu, Tokyo 135-8548, Japan; 2 College of Engineering, Shibaura Institute of Technology, 3-7-5 Toyosu, Tokyo 135-8548, Japan; tipporn@shibaura-it.ac.jp (T.L.); doly@shibaura-it.ac.jp (M.S.); 3 MIRAI Technology Institute, Shiseido Co., Ltd., 1-2-11 Takashima, Nishi-ku, Yokohama 220-0011, Kanagawa, Japan; toshi@shiseido.com (T.M.); keiko.tagai@shiseido.com (K.T.)

**Keywords:** positive affect, heart rate variability, EEG

## Abstract

Positive affect comprises distinct affective states that differ in arousal level, such as high-arousal positive affect (HAPA) and low-arousal positive affect (LAPA), which have been shown to be associated with different effects and effective contexts. In studies of positive affect, it is therefore important not only to assess overall positivity but also to distinguish between different types of positive affect. Existing assessments rely mainly on self-reports, which may be unreliable for individuals with limited self-report abilities. The aim of this study was to examine whether physiological indices can discriminate between HAPA and LAPA. Participants were presented with eight video stimuli designed to elicit either HAPA or LAPA, and self-report measures were used as manipulation checks to define the affective conditions, while heart rate variability (HRV) and electroencephalography (EEG) were recorded. HRV indices did not show significant differences between the two affective conditions. In contrast, analyses of EEG relative power revealed significant differences between the HAPA and LAPA conditions. These findings demonstrate that, under the present experimental conditions, physiological differences between low- and high-arousal positive affect can be captured in EEG signals using relative power, a simple and reproducible analytical index, whereas no such differences were observed in HRV indices.

## 1. Introduction

### 1.1. Background

Stress and mental health problems represent major public health challenges in con-temporary society. According to the World Health Organization (WHO), as of 2021, nearly one in seven people around the world were living with a mental dis-order [1,2]. Multinational survey data further indicate that emotional stress has worsened in many countries since 2008, suggesting that stress is not confined to specific regions [3]. Chronic stress has also been reported to be associated with an increased risk of cardiovascular diseases such as hypertension and coronary artery disease, as well as sleep disturbances, depression, and anxiety [4,5]. Thus, chronic stress may impair both physical and psychological functioning. In this regard, positive affect, an emotional state associated with stress reduction and health promotion, has recently garnered increasing attention. It has been suggested that positive affect may play an important role in enhancing both psychological and physical health [6,7,8].

Positive psychology is a research field that focuses on positive affect and positive personal characteristics, with the aim of cultivating and enhancing them [9]. The introduction of positive psychology broadened recognition of positive affect as a domain requiring systematic and integrated scientific understanding, leading to increased academic attention [10]. As research on positive affect expanded, associations between positive affect and various aspects of human behavior and health have become increasingly evident. According to Fredrickson and Levenson, positive emotions broaden individuals’ momentary thought–action repertoires, and this broadening, over time, contributes to the development of enduring personal skills and resources [11]. It has also been shown that individuals’ momentary cognitive capacities, such as the scope of attention, broaden when they are primed with positive affect [12]. Moreover, higher self-reported levels of positive emotion have been shown to be associated with greater ability to understand others’ emotions, a skill that is essential for building cooperative relationships [13,14].

Based on these findings, various positive psychology interventions (PPIs) that utilize positive affect have been proposed as methods for applying positive affect. PPIs practically apply positive affect to enhance individual well-being and to provide psychological support. Research involving educators has shown that fostering positive affect in instructional environments can serve as an important psychological foundation for adopting innovative teaching practices [15]. In clinical psychology, interventions that enhance gratitude have been shown to reduce depression and anxiety, and techniques that strengthen positive traits contribute to the treatment of emotional disorders [16]. Moreover, Tarrier and colleagues reported an intervention in which individuals with PTSD improved social engagement and self-efficacy through helping others [17]. In addition, PPIs seem promising for enhancing cognition and brain functioning in older adults with cognitive decline [18]. Thus, research utilizing positive affect as an intervention shows promise across a wide range of domains, including settings such as education and caregiving.

To appropriately implement and evaluate PPIs, it is essential to verify whether the intended emotion was sufficiently elicited. This is because insufficient verification of emotion induction can undermine the validity of the intervention effects and the theoretical assumptions. However, existing methods for positive affect have not yet established evaluation approaches other than self-report [19]. The target populations of PPIs include not only healthy individuals but also older adults and people with mental disorders; however these individuals may have difficulty accurately recognizing and self-reporting their own emotional states [20,21,22,23]. Therefore, assessment methods that rely solely on self-report, as commonly used in previous emotion research, may not provide sufficient reliability in assessing emotional states. For these reasons, there is a need for methods to assess positive affect without relying solely on subjective evaluations.

### 1.2. Related Work and Issues

For indicators used to assess positive affect in individuals for whom emotional self-report is difficult, two key points should be considered.

The first point concerns whether the indicator can distinguish between different types of positive affect. The positive affect is not a uniform construct; it encompasses multiple distinct emotional states, each of which is considered to have different effects [24,25]. Therefore, in order to utilize appropriate positive affect in appropriate contexts, indicators that can distinguish between different positive affective states are required. The second point is the need to examine indicators that can be obtained relatively easily even outside controlled experimental settings. This is because it is necessary to consider future applications in educational and caregiving settings, where the use of positive affect is expected [15,16,17,18].

In the following subsections of Section 1.2, studies related to the aforementioned considerations are presented in order to organize and clarify the issues addressed in this study.

#### 1.2.1. Category of Positive Affect

Positive affect is not a unitary construct; it is considered to be distinguishable into two categories: High-Arousal Positive Affect (HAPA) and Low-Arousal Positive Affect (LAPA) [25,26]. HAPA refers to high-arousal positive affect accompanied by emotions such as happiness, excitement, and enthusiasm, whereas LAPA refers to low-arousal positive affect characterized by feelings of calmness and relaxation [25]. For example, McManus et al. compared how HAPA and LAPA contribute to predicting mental health and well-being, and showed that LAPA may have stronger predictive power than HAPA [25]. In addition, Gilbert et al. provided supporting evidence for the view that HAPA promotes performance enhancement, whereas LAPA promotes a sense of safety [27].

Furthermore, Barrett et al. have noted that distinguishing between HAPA and LAPA may be particularly useful for individuals with low emotional granularity, who have difficulty differentiating their own emotional states [28]. This is because the cognitive processes associated with positive affect are known to strengthen and enhance coping abilities, yet individuals with low emotional granularity find it difficult to differentiate and recognize these emotional states. Painter et al. have also shown that excessive HAPA states may have adverse effects for individuals with bipolar disorder [29]. Accordingly, interventions for bipolar I disorder emphasize not only modifying positive and negative emotions but also regulating arousal states [29].

Thus, rather than treating HAPA and LAPA as a single category of positive affect, it is considered important to identify them as distinct categories. This is also relevant from an applied perspective. However, in positive psychology, methods for emotion assessment that can distinguish between HAPA and LAPA have not been adequately investigated.

#### 1.2.2. Assessing Emotional States Using Physiological Indices

As an alternative approach to address the limitations of self-reported emotion assessment, the use of physiological indices for assessing emotional states has recently attracted increasing attention. Physiological indices refer to measures obtained by recording physiological responses such as brain activity, heart rate, respiration, and electrodermal activity. Because these indices are derived from measurements of physiological responses, they can be obtained continuously over time without requiring any conscious effort from the individual. Compared with self-reported emotion assessments, physiological indices do not require participants to provide responses for each stimulus [30,31,32,33,34]. Owing to these characteristics, emotion assessment based on physiological indices has become increasingly prominent in research areas such as affective computing and human–robot interaction. The effectiveness of emotion assessment using physiological indices has been demonstrated in multiple studies [30,31,32,33,34].

Several studies have applied the utility of physiological indices to research on positive affect [35,36]. Among them, Shiota et al. demonstrated, using autonomic nervous system indices, that positive affect includes five distinguishable emotional states: anticipatory enthusiasm, attachment, nurturant love, amusement, and awe [35]. In addition, Hu et al. investigated the relationship between electroencephalography (EEG) and various discrete positive emotions [36]. Through correlation analyses between EEG and ten discrete positive emotions (awe, gratitude, hope, inspiration, pride, amusement, joy, interest, love, and serenity), they demonstrated differences in how these emotions relate to EEG activity [36]. Based on the related studies presented, relationships between positive affect and physiological indices have been reported.

#### 1.2.3. Issues

As discussed in Section 1.2.2, physiological indices may also be applicable to the assessment of positive affect in target populations for whom self-report is difficult. On the other hand, we considered that two issues exist as a preliminary step before aiming to assess positive affect using physiological indices. On the first issue, it is necessary to examine whether physiological indices exist that reflect differences between two distinct categories of positive affect. As described in Section 1.2.1, positive affect is considered to be broadly classified into two categories, namely HAPA and LAPA. Moreover, these two categories of positive affect have been reported to exhibit different effects [25,26]. Therefore, clarifying indices that reflect differences between categories of positive affect may enable the assessment of appropriate positive affective states according to specific application contexts. On the second issue, it is necessary to examine more appropriate physiological indices with a view toward practical use across a wide range of settings outside laboratory environments, such as education and caregiving contexts. However, previous research has not identified differences in physiological indices between the two categories of positive affect, HAPA and LAPA, nor has it examined physiological indices with consideration for practical applications.

## 2. Purpose and Proposal

### 2.1. Purpose

Considering the above points, this study aims to identify physiological indices that can effectively distinguish. In particular, based on the issues discussed in Section 1.2, physiological indices are examined and evaluated with consideration for applications outside laboratory environments. The proposed method for achieving this objective is described in Section 2.2.

### 2.2. Proposal Method

#### 2.2.1. Hypothesis and Experimental Design

To achieve the aim of this study, we tested the following hypothesis to clarify an approach for identifying physiological indices that can distinguish between HAPA and LAPA in emotion assessment:

The conditions under which LAPA and HAPA states can be distinguished based on self-reported emotion assessments will be identified, and under those conditions, statistically significant differences will also be observed in the recorded physiological indices.

This approach is based on the premise that identifying conditions under which positive affect can be distinguished through self-reported emotion assessments, and then confirming that physiological indices also distinguish these conditions, indicates that physiological indices capture differences in emotional states.

Appropriate questionnaire items for self-reported emotion assessments of HAPA and LAPA were selected, and relevant physiological indices were investigated. The selection of questionnaire items is presented in Section 2.2.2, and the examination of physiological indices is presented in Section 2.2.3. An experiment using video stimuli designed to elicit positive affect was then designed and conducted, and the effectiveness of the proposed approach was demonstrated through analysis of the experimental results. In the analysis, (i) a manipulation check based on self-reported emotion assessments was conducted to identify which stimuli elicited HAPA and LAPA, thereby defining the two conditions: the HAPA condition and the LAPA condition. (ii) physiological indices showing statistically significant differences between the HAPA and LAPA conditions were identified.

Following this procedure, we considered that it would be possible to identify physiological indices that reflect differences between distinct positive affective states. In this experimental design, positive affective states are labeled based on self-report, followed by analyses using physiological indices. Therefore, in this study, data are collected from healthy adults who are considered capable of reporting their own emotional states as experimental participants.

#### 2.2.2. Questionnaire Items

In this study, to distinguish whether the elicited positive affect corresponded to HAPA or LAPA, questionnaire items were selected with reference to four previous studies and assessment scales. First, we referred to the review paper by McManus et al., which summarized emotion terms widely used to assess HAPA and LAPA across 150 studies [25]. This was used as a reference for selecting emotion terms commonly used in the evaluation of LAPA and HAPA [25]. Second, we referred to the PANAS-X proposed by Watson et al. [37]. PANAS-X is an extension of the original PANAS scale, which is regarded as one of the most widely used scales for the assessment of positive affect [19]. Although the items included in the original PANAS were biased toward assessing HAPA, PANAS-X incorporates items associated with LAPA, including “calm,” “relaxed,” and “at ease.”. Third, we referred to the Japanese Multi-Dimensional Mood State Scale, which provides multidimensional assessment items for emotional states [38]. This scale was adopted because it contains items assessing a wide range of emotions in Japanese, from negative to positive affect. Fourth, we referred to the scale proposed by Matsubara et al. [39]. This scale evaluates positive affect in Japanese individuals by dividing emotions such as calmness and excitement into four factors.

Based on these references, six items were selected such that higher scores indicate LAPA and lower scores indicate HAPA. In the actual user evaluation, participants rated each item on a five-point scale (1–5). The total score, ranging from 6 to 30, was used for self-reported emotion assessment.

#### 2.2.3. Physiological Indices Selection

In general, physiological responses associated with human emotions are considered to arise primarily from the autonomic nervous system (ANS) and the central nervous system (CNS) [30,40,41]. The ANS is a general physiological regulatory system responsible for peripheral functions [40].

The ANS consists of the sympathetic nervous system, which is associated with physiological activation, and the parasympathetic nervous system, which is associated with relaxation. The sympathetic nervous system prepares the body for stress-related responses, whereas the parasympathetic nervous system functions in the opposite manner, promoting a calm, low-arousal physiological state [42]. The CNS is composed of the brain and spinal cord. The brain controls various functions of the body, and changes in its electrical activity are translated into diverse behaviors and emotional responses. Thus, although activities of the two neural systems have been shown to be related to human emotional states, no previous studies have clarified whether the physiological indices that differentiate positive affect—particularly the two distinct categories of HAPA and LAPA—reflect activity of the ANS or the CNS. Accordingly, in the present study, indices reflecting the activity of each neural system are selected and evaluated without restricting the analysis to indices associated with only one of the two systems.

(1) HRV

Physiological indices used to evaluate ANS activity include electrodermal responses and cardiovascular responses [30]. Among these, HRV is considered one of the most commonly used indices of ANS activity [43]. HRV is defined as the variability in the intervals (in milliseconds) between consecutive heartbeats (RR intervals). To obtain these heartbeat intervals, continuous measurement of heart rate is required, typically using electrocardiography (ECG). HRV is regarded as a noninvasive physiological technique that enables measurement of both sympathetic and parasympathetic activity within overall ANS responses, making it suitable for emotion-related research [43].

Several analytical approaches exist for HRV. Among them, time-domain indices, which quantify the variability of interbeat intervals (IBI) using statistical measures, are expected to be applicable in fields such as affective computing [34]. Time-domain indices quantify IBI variability using statistical metrics [44]. SDNN is considered an index reflecting both sympathetic and parasympathetic activity, whereas RMSSD and pNN50 are considered indices reflecting parasympathetic activity [42]. Accordingly, in this study, SDNN, RMSSD, and pNN50 were selected as physiological indices for evaluation (Table 1).

(2) EEG

The central nervous system (CNS) is composed of the brain and spinal cord. The brain controls various functions of the body, and changes in its electrical activity are translated into diverse behaviors and emotional responses. Methods for measuring CNS activity include EEG, functional magnetic resonance imaging (fMRI), and positron emission tomography (PET) among others [46,47]. Among the physiological indices used to evaluate the CNS in emotion research, EEG is one of the most widely used methods [40]. EEG records the brain’s electrical activity, and because of its high temporal resolution, researchers are able to observe changes that occur in response to emotional stimuli [48,49,50]. Furthermore, EEG is considered a favorable method for investigating neural responses to emotional stimuli owing to its noninvasiveness, rapid acquisition, and relatively low cost [51,52,53]. Owing to these advantages, EEG is considered to be one of the most widely used neuroscientific tools among researchers [54]. Furthermore, in recent years, various consumer-grade EEG devices have been developed, and they are considered to enable data collection outside traditional laboratory environments [55].

These research involve extracting time, frequency, time-frequency, and non-linear features from preprocessed EEG signals [56]. The power spectral density (PSD) analysis is a classical frequency-domain method that can effectively quantify the energy distribution characteristics in different frequency bands [57]. The frequency domain features have a better anti-interference to noise and can reflect the details of each component of the signal [58]. Therefore, the PSD, are widely extracted in physiological emotion calculation [58]. The reliability of the frequency features, particularly power in standard frequency bands, has been well studied, with early studies confirming the reliability and stability of power across different frequency bands [59,60,61]. EEG signals are classified into multiple frequency bands based on their spectral components [62]. These are commonly divided into the delta band (1–3 Hz), theta band (4–7 Hz), alpha band (8–12 Hz), beta band (13–30 Hz), and gamma band (>30 Hz). Each band is considered to reflect different functional states of the brain. Delta activity is associated with sleep, deep relaxation, cortical inhibition, and low-arousal emotional processing. Theta activity is related to attentional control, working memory, and internal cognitive processes. Alpha activity is associated with relaxed wakefulness and inhibition of external stimuli, whereas beta activity increases during focused attention. Gamma activity has been reported to be associated with perceptual integration, higher-order cognitive processing, and local neural synchrony [48,63].

Although delta activity is prominent during sleep and deep relaxation, it is known to be highly susceptible to artifacts (e.g., body movements and eye movements) during wakefulness. Therefore, to evaluate neural activity under emotion elicitation tasks more appropriately, this study focused on the theta–beta bands as the primary targets of analysis (Table 2).

In addition, this study uses relative power rather than raw band power for analysis. The PSD can vary with the individuals, time, and system [64]. Therefore, using relative power that the power at a specific frequency relative to the total power is more technically sound to ensure the power level variation measurement of the concerned band with respect to the full band power in case of EEG signals [65,66]. Accordingly, in this study we selected to use relative power.

### 2.3. Contribution and Structure of This Study

The main contribution of this study is the identification of physiological indices that can capture the difference between HAPA and LAPA states without relying solely on self-reported emotion assessments. To our knowledge, no previous research has distinguished HAPA and LAPA states using physiological indices. Identifying physiological indices that are effective for distinguishing HAPA and LAPA states has the potential to expand future applications, such as evaluating these states in individuals for whom self-reporting is difficult and verifying the effects of PPIs.

Although previous studies have attempted to distinguish discrete positive emotions or have theoretically examined the classification of positive affect based on arousal levels, systematic investigations of HAPA and LAPA states using physiological indices are, to the best of our knowledge, scarce. This gap represents an important missing link in both advancing our understanding of the structure of positive affect and improving the verification of emotion elicitation in PPIs. The present study aims to address this gap.

This paper is structured as follows. Section 3 describes the experiment conducted in this study. Section 4 presents the analytical methods. Section 5 discusses the results and provides implications for the distinction and assessment of HAPA and LAPA. Finally, Section 6 concludes the study.

## 3. Experimental Method

### 3.1. Stimuli

In this study, video stimuli were used to elicit positive affect. In research on emotion assessment using physiological indices, experimental procedures typically present stimuli such as images, sounds, and videos to elicit emotions, and then obtain self-reported emotion assessments and record physiological signals. Stimuli used in this line of research commonly include images, sounds, and videos [67]. Among these, video stimuli, which combine both visual and auditory elements, have been shown to be effective for emotion elicitation [36]. Therefore, video stimuli were adopted in this study.

The videos used in this study were those previously used in our previous experiments [68]. Eight videos with audio were created by purchasing sound-free footage from Shutterstock (https://www.shutterstock.com/ (accessed on 8 January 2026)) and combining it with background audio downloaded from a free sound-source website (https://dova-s.jp/ (accessed on 8 January 2026)). Each of the eight videos was assigned an ID (VideoID-1 to VideoID-8). VideoID-1 to VideoID-4 contained fast-tempo music and dance performances and were expected to elicit HAPA. VideoID-5 to VideoID-8 included slow and calm music, as well as scenes such as affectionate behavior of dogs and natural landscapes, which were expected to elicit LAPA. This configuration enabled the comparison of self-reported emotion assessments and physiological responses to positive affect with different arousal states.

### 3.2. Measuring Instruments

**Figure 1:**
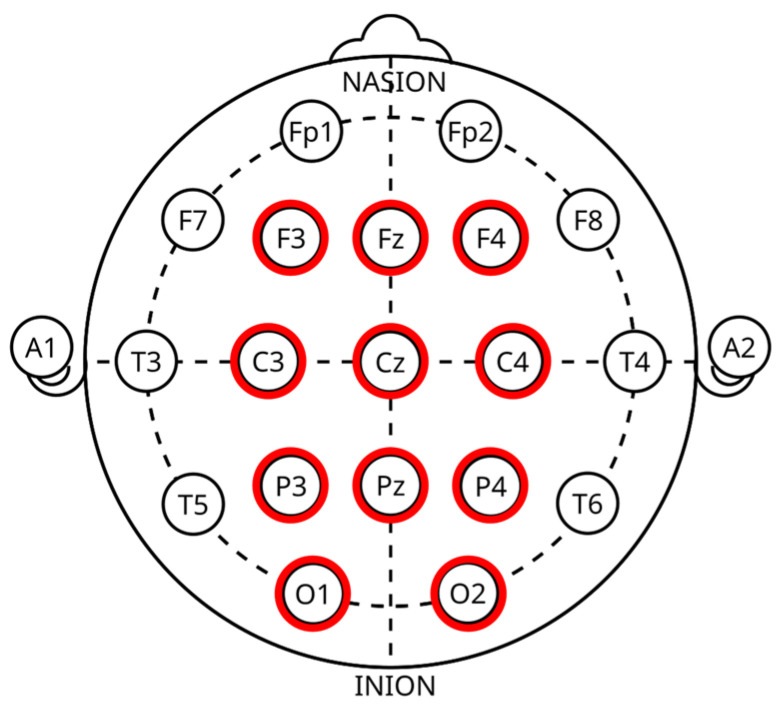
10–20 international electrode positioning [70].

Physiological signals were recorded using the Polymate V AP5148 (Miyuki Giken Co., Ltd., Tokyo, Japan). Signal data were collected on a recording PC using the dedicated software AP-Monitor (Ver 6). The sampling rate was set to 1000 Hz. To reduce electrical noise across all channels, the ground electrode was placed on the posterior neck. A photosensor was connected as a trigger to minimize timing discrepancies between stimulus presentation and physiological recording. ECG was recorded using a three-electrode configuration, with electrodes placed on the left abdomen and below the mid-clavicle, approximating lead II. This configuration provides high R-wave detection accuracy and is suitable for HRV analysis [42]. EEG signals were recorded using an EEG cap based on the international 10–20 system [69]. Eleven electrodes were used: F3, Fz, F4, C3, Cz, C4, P3, Pz, P4, O1, and O2, as shown in Figure 1. Electrode impedance was maintained below 20 kΩ during recording.

### 3.3. Experimental Setup and Procedure

**Figure 2:**
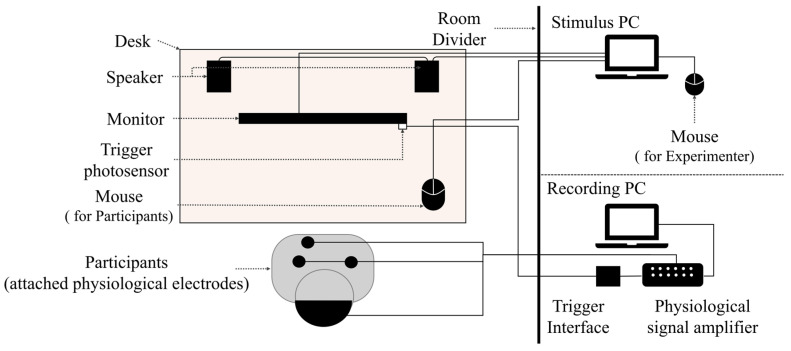
Experimental setup.

The experiment was conducted individually in a quiet, private room. To present video stimuli and questionnaires to the participants, a 27-inch monitor was placed at the center of the desk where each participant was seated, and stereo speakers were positioned on both sides. These output devices were connected to a computer used for stimulus presentation. The computer was placed behind a partition panel to prevent it from being visible to the participants. A mouse was placed on the desk for participants to use when responding to the questionnaires. Participants were permitted to use the mouse only during questionnaire responses. The experimental setup is shown in Figure 2.

**Figure 3:**
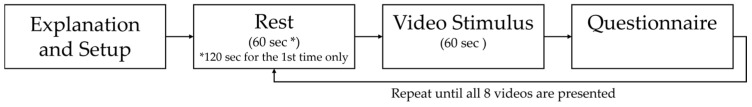
Experimental procedure. * Rest period was set to 120 s only for the first trial.

The experimental procedure used in this study is shown in Figure 3.

Before the experiment began, the experimenter provided participants with a verbal explanation of the study and obtained their informed consent. Sensors were then attached to participants who provided consent, and it was confirmed that physiological signals were being recorded properly. Afterward, participants completed a practice session using a training display, and once all adjustments were completed, the actual experiment began. In the actual experiment, the sequence of “pre-stimulus rest,” “stimulus presentation,” and “questionnaire response” was repeated eight times for the eight video stimuli. The rest period was set to 120 s only for the first trial to allow participants to settle into the experimental context, and to 60 s for the remaining trials to minimize carryover effects from the preceding stimulus.

To avoid order effects, the presentation order of the eight videos was randomized for each participant. The questionnaire was displayed on the computer screen after each video was viewed. At the top of the screen, the following instruction was shown: “After watching the video, please choose the number that best represents how you feel right now.” The selectable responses ranged from 1 to 5, and the meaning of each number was displayed at the top of the screen: 1 = “Does not apply at all,” 2 = “Applies slightly,” 3 = “Neither,” 4 = “Applies somewhat,” and 5 = “Applies very much.” In addition, all questionnaire items were randomized to prevent order effects and reduce potential biases toward specific factors.

### 3.4. Participants

54 participants (aged 20–40 years; 35.0 ± 8.5 years; 50% female) who provided informed consent took part in the experiment. All participants were native Japanese speakers and were confirmed to have no significant visual or auditory impairments. Participants were also screened to ensure they had no history of neurological, psychiatric, or cardiovascular disorders. In addition, all participants were confirmed to be non-smokers with no smoking history. All participants were also confirmed to be right-handed. Only individuals who met all of these criteria were included in the study.

Only individuals who met all of these criteria were included in the study. Participants were informed that their participation was voluntary and that they had the right to withdraw at any time.

4 participants were excluded from the analysis. 1 participant was excluded because their questionnaire responses indicated that they might not have engaged with the task appropriately. The remaining 3 participants were excluded due to excessive noise in their EEG signals. The number of participants by age group and gender is shown in Table 3.

The study procedures were approved by the Ethics Committee of Shiseido (Approval number: C10374)., and all participants signed an informed consent form.

## 4. Data Analysis Method

### 4.1. Physiological Signal Preprocessing and Index Computation

This section describes the physiological indices used in this study, the sensors employed to record physiological signals, and the procedures for preprocessing the obtained data and computing physiological indices. ECG and EEG signals were preprocessed, and physiological indices were computed, after which baseline correction was applied to construct the final dataset used for statistical analysis.

#### 4.1.1. ECG Preprocessing and HRV Index Computation

The recorded ECG signals were preprocessed using a zero-phase bandpass filter to improve R-peak detection accuracy, following the standard methods implemented in the Python library NeuroKit2 (v.0.2.10) [45,71]. After preprocessing, the ECG signals were segmented into 60 s intervals corresponding to each stimulus presentation and the initial resting period for each participant. For each interval, R-peaks were detected, and HRV indices were computed based on the resulting interbeat interval information. In this study, three HRV indices listed in Table 1 were computed using the 60 s periods from the initial rest condition and each stimulus presentation condition [42].

#### 4.1.2. EEG Preprocessing and Index Computation

EEG preprocessing was first performed using EEGLAB (v.2023.1) running on MATLAB R2023a, following widely adopted standard preprocessing procedures in EEG research [72,73]. After applying 1 Hz high-pass, 100 Hz low-pass, and 50 Hz notch filters, the average signal across all EEG channels was then computed and subtracted from each channel to perform average re-referencing. Next, independent component analysis (ICA) based on the extended-Infomax algorithm was performed, and the resulting components were classified using the ICLabel plugin [74]. This ICA-based preprocessing approach has been commonly adopted in EEG studies, including affective computing research and studies employing dynamic visual stimuli, where ocular and muscle artifacts are unavoidable [75,76,77]. Components with a probability below 0.7 of being classified as neural activity were automatically rejected. After component rejection, missing channel information was interpolated using spherical interpolation to restore the original channel configuration.

To compute the EEG indices described in the Proposal Method, the preprocessed EEG data were converted for use in a Python environment and analyzed using MNE-Python (v.1.6.1) [78]. The data exported from EEGLAB were imported, and 60 s epochs were constructed for each corresponding event. Each epoch was divided into non-overlapping 2 s windows (256 Hz × 2 s = 512 samples), and Welch’s method was applied to each window to computed the PSD. Subsequently, band power was obtained by integrating the PSD within the theta (4–7 Hz), alpha (8–12 Hz), and beta (13–30 Hz) bands. The total power in the 1–30 Hz range was also computed to derive relative power.

### 4.2. Data Normalization Method

Because the data collected from participants can vary substantially due to individual differences in physiological signals, the data were normalized before further analysis following the procedure described by Laohakangvalvit et al. [79]. Specifically, the mean value of each physiological index was adjusted by subtracting the participant’s baseline mean obtained during the resting period. Consequently, subsequent analyses considered only the change from baseline in each physiological index during the video stimuli. The resting period was chosen as the baseline because it was conducted at the beginning of the experiment and was not influenced by any prior stimuli. Therefore, this condition was used as the participant’s neutral physiological baseline.

### 4.3. Manipulation Check with Self-Reported Emotion Assessment

In this experiment, to verify whether the HAPA and LAPA stimuli elicited the intended emotional responses, self-reported emotion assessments for each stimulus were obtained immediately after its presentation. The self-reported emotion assessments were analyzed using a one-way within-subjects repeated-measures ANOVA with stimulus as the factor. When normality was violated, the nonparametric Friedman test was applied. When the Friedman test indicated a significant effect, pairwise Wilcoxon signed-rank tests were conducted, followed by Holm correction for multiple comparisons. Effect sizes (r) were computed for each pair. These analyses were used to statistically confirm whether the stimulus manipulation functioned as intended.

### 4.4. Comparison of Physiological Indices

Based on the results confirmed in the manipulation check, participant-level mean values were calculated for the HRV and EEG indices, and paired comparisons between conditions were conducted. For HRV indices, normality for each condition was assessed using the Shapiro–Wilk test. When normality was satisfied, paired *t*-tests were applied; when not, Wilcoxon signed-rank tests were used. For EEG, a two-way repeated-measures ANOVA with condition (2) and channel (11) as factors was conducted for each frequency band. When the residuals violated normality, an Aligned Rank Transform (ART) ANOVA was used [80]. The aim of this study was to identify physiological indices capable of distinguishing between HAPA and LAPA. Accordingly, statistical testing and multiplicity control were performed separately within each frequency band. For frequency bands showing significant main effects or interactions, paired comparisons between conditions were conducted as post hoc tests for each channel, with Holm correction applied for multiple comparisons. The overall significance level was set to 0.05.

## 5. Results and Discussion

### 5.1. Manipulation Check Statistical Comparison Among Stimuli for Manipulation Check and Select Stimuli

**Figure 4:**
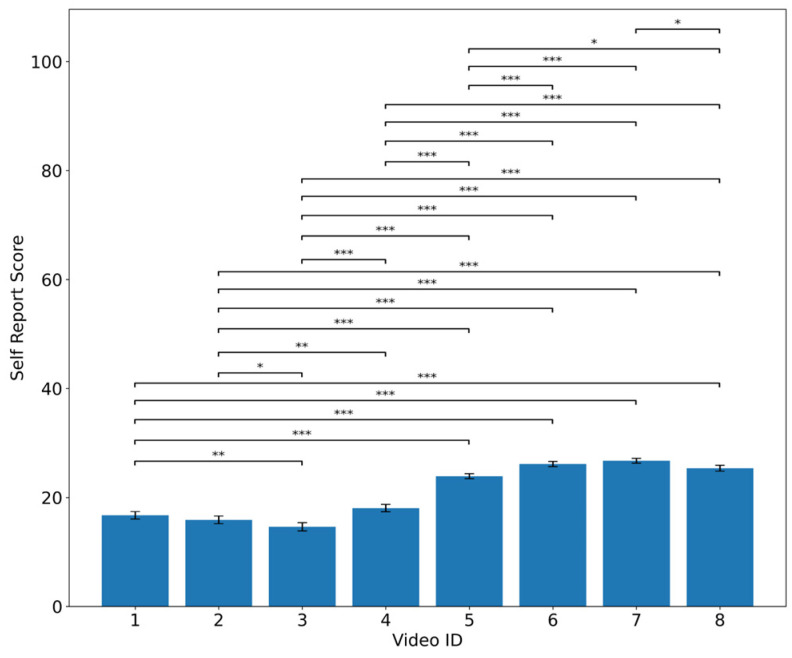
Mean self-reported emotion assessment scores with Wilcoxon signed-rank test results. * *p* < 0.05, ** *p* < 0.01, *** *p* < 0.001.

The Friedman test conducted on the self-reported emotion assessments revealed a significant main effect (χ^2^(7) = 243.653, *p* < 0.001, Kendall’s *W* = 0.696; *n* = 50, *k* = 8). As post hoc analyses, Wilcoxon signed-rank tests were conducted for all stimulus pairs, and Holm correction was applied. The results showed that the stimuli intended to represent the LAPA condition (VideoID-5 to VideoID-8) consistently yielded significantly higher self-reported emotion assessment scores than the HAPA stimuli (VideoID-1 to VideoID-4), with all comparisons remaining significant after correction (*p* < 0.001). The mean self-reported scores for each stimulus are shown in Figure 4. The *p*-values and confidence intervals for each stimulus pair are shown in Table A1.

The effect sizes (*r*) for all Wilcoxon signed-rank test pairs among the eight stimuli are presented in Table 4.

Consistently large effect sizes (r ≈ 0.8 or higher) were observed between the HAPA stimulus group (VideoID-1 to VideoID-4) and the LAPA stimulus group (VideoID-5 to VideoID-8), indicating that the intended emotional manipulation functioned as expected. Based on these results, we next examined whether the differences identified in the self-reported emotion assessments were also reflected in the physiological indices. To this end, the HAPA and LAPA groups were treated as two conditions for comparative analysis of the physiological indices.

### 5.2. Physiological Analyses

As described in Section 2.2.3, this study selected HRV as indices reflecting the ANS activity and relative power as indices reflecting the CNS activity, and evaluated whether each index exhibited significant differences between the HAPA and LAPA conditions. The corresponding results and discussion are presented in Section 5.2.1 and Section 5.2.2, respectively.

#### 5.2.1. HRV

First, the HRV indices were compared between the HAPA and LAPA conditions. The results of the Wilcoxon signed-rank tests are presented in Table 5. For none of the HRV indices (RMSSD, SDNN, pNN50) was a significant difference observed between the LAPA and HAPA conditions (*p* > 0.05). The effect sizes were all small (RMSSD: *r* = 0.07; SDNN: *r* = 0.24; pNN50: *r* = 0.09), indicating that the emotional manipulation confirmed through self-reported emotion assessments did not produce pronounced changes in HRV. These findings suggest that HRV indices may not serve as physiological indices that reflect the differences between HAPA and LAPA states.

Several possible explanations can be considered for the absence of significant differences in HRV indices. One possible explanation is that the duration of stimulus presentation may have been too short. In this experiment, the stimulus presentation duration was relatively short, at 60 s. Previous studies have suggested that the HRV indices used in this study may allow the calculation of reliable values within a short duration of 60 s [81,82]. On the other hand, prior studies have suggested that stimuli eliciting positive affect elicit relatively smaller physiological responses compared with stimuli eliciting negative affect [83]. Based on these considerations, when HRV indices are used to evaluate responses to stimuli eliciting positive affect, a measurement duration longer than 60 s may be required.

As a second possibility, the selected HRV indices may not reflect differences in arousal levels within positive affect, such as those between HAPA and LAPA. The HRV indices selected in this study are associated with parasympathetic nervous system activity within the ANS, and did not include indices that purely reflect sympathetic nervous system activity [42]. Therefore, indices reflecting sympathetic nervous system activity within the ANS may show differences between HAPA and LAPA. Moreover, prior work by Shiota et al. has demonstrated differences among five distinct positive affects using ANS-related indices [35]. Based on the above discussion, it may be possible to distinguish differences among positive affects that are categorized according to criteria other than arousal level. Accordingly, although HRV indices were selected in this study as one type of ANS-related index, it should be noted that the present results do not necessarily indicate that ANS activity fails to reflect differences between positive affective states with different arousal levels, such as HAPA and LAPA.

#### 5.2.2. EEG

For EEG activity, a two-way repeated-measures analysis with condition (2) and channel (11) as factors was conducted to test differences across frequency bands between the HAPA and LAPA conditions. Because the residuals violated normality in all frequency bands, an Aligned Rank Transform (ART) ANOVA was applied to all bands. The results are presented in Table 6.

According to Table 6, significant main effects of condition and channel were observed in the theta and beta bands. These results suggest the possibility of overall differences in EEG activity between conditions. In the alpha band, significant main effects of condition and channel, as well as a significant interaction, were observed. This indicates that differences between conditions were present, but that their patterns may vary across channels. Therefore, paired comparisons between the HAPA and LAPA conditions were conducted for each channel within all the three frequency bands, and Holm correction was applied for multiple comparisons. The channels that showed significant differences between conditions in each frequency band are summarized in Table 7.

**Figure 5:**
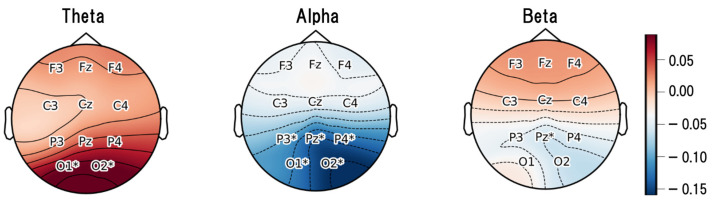
Mean differences between the HAPA and LAPA conditions, with significant channels marked by asterisks (*).

In the theta band, significant differences were observed at the occipital channels O1 and O2. In the alpha band, significant differences were found across a broad region from occipital to parietal areas, including O1, O2, P3, P4, and Pz. In contrast, in the beta band, a significant difference was observed only at the parietal channel Pz. These results indicate that the differences between conditions were primarily concentrated in the occipital–parietal regions. To further examine the differences between the HAPA and LAPA conditions, the difference between their mean values is presented in Figure 5. Channels that showed significant differences in the frequency bands listed in Table 7 are marked with asterisks (*).

In the theta band, the LAPA condition showed significantly higher activity than the HAPA condition at the occipital sites O1 and O2. Theta-band activity is associated with internally directed processes such as memory retrieval, emotional recollection, and meditation, and has been suggested to suppress external stimulus processing while supporting self-referential cognitive states [84,85]. Aftanas and Golocheikine reported that theta activity increases during states of internalized attention and emotional absorption in meditation. The present results are functionally consistent with these findings and suggest that the LAPA state involves enhanced internal attention and a more static arousal state.

In the alpha band, relative power decreased under the LAPA condition across occipital and parietal–occipital regions, including P3, P4, Pz, O1, and O2. Alpha activity is known to be involved in arousal regulation and attentional control. A decrease in its proportion indicates relatively increased neural activity related to sensory processing and emotional recollection [86,87]. Therefore, the present findings may reflect heightened internal attention in the LAPA state, characterized by reduced attention to external stimuli and greater focus on one’s emotional experience.

Furthermore, in the beta band, a decrease in relative power was observed at the mid-parietal site Pz. Beta activity has been reported to be involved in maintaining arousal and supporting cognitive control [88]. The reduced beta relative power observed in this study may reflect decreased cognitive tension and control in the LAPA condition, indicating a more relaxed state. This pattern is also consistent with prior reports indicating that beta-band activity decreases during emotional relaxation or lowered arousal [89,90].

However, considering that the observed EEG differences were predominantly localized to occipital and parietal regions, which are strongly involved in visual processing and visuospatial attention, these patterns should be interpreted as group-level neural differences observed under visually dynamic and attentionally demanding stimulation, rather than as definitive neural markers of internal positive affect independent of sensory and attentional processes [91,92].

### 5.3. Contributions

This study focused on the distinction between HAPA and LAPA within positive affect using physiological indices. Using video stimuli to elicit positive affect, self-reported emotion assessments were obtained while ECG and EEG signals were recorded, and differences in the physiological indices between the LAPA and HAPA states were analyzed. As a result, although no significant differences were found in the HRV indices, differences between conditions were identified in the EEG indices. Under the LAPA condition, relative power increased in the theta band in occipital regions, while alpha-band relative power decreased in occipital–parietal regions, and beta-band relative power decreased at parietal sites.

A key contribution of this study is that it clarified the physiological characteristics of HAPA and LAPA in terms of EEG relative power. It was also demonstrated that physiological differences between LAPA and HAPA can be detected using relative power, a simple and reproducible analytical index. The results showed that even a relatively simple analysis based solely on relative power is sufficient to identify statistical differences between LAPA and HAPA. These findings hold significance for establishing physiological-index-based emotion assessment as a more generalizable and reproducible approach. Using video stimuli to elicit positive affect, self-reported emotion assessments were obtained while ECG and EEG signals were recorded, and differences in the physiological indices between the LAPA and HAPA states were analyzed.

### 5.4. Limitation and Future Work

This study has several limitations. First, video stimuli were used to elicit positive affect. Compared with static images or auditory stimuli, video stimuli contain richer visual information and inherently engage dynamic attentional and perceptual processes. Therefore, some of the EEG differences observed in occipital–parietal regions may reflect not only emotional processing but also attentional load and sensory integration associated with visually rich stimuli. Accordingly, the present EEG findings should be interpreted as reflecting emotion-related neural activity under conditions that involve substantial visual attention, rather than as purely emotion-specific neural signatures. To further disentangle emotional processing from attentional and perceptual influences, future studies should systematically compare different stimulus modalities, such as static images or auditory stimuli, under controlled experimental conditions.

Second, although HAPA and LAPA were defined with reference to previous studies, the present study did not directly examine their correspondence to high-valence low-arousal states within dimensional models of emotion, which represents a limitation. The self-reported emotion assessments used in this study do not allow for quantitative identification of their positions within the two-dimensional affective space. Therefore, caution is required when interpreting how the HAPA and LAPA states correspond to regions within dimensional models of emotion. In future work, in order to evaluate how affective states defined as HAPA and LAPA can be positioned within the two-dimensional model of emotion, it is considered necessary to conduct comparative analyses between HAPA and LAPA scores and questionnaire-based assessments, such as the Self-Assessment Manikin, which enable the evaluation of human affective states in accordance with the two-dimensional emotion model [93].

Third, the EEG analyses in this study were limited to relative power, and other advanced EEG measures such as event-related dynamics, functional connectivity, and cross-frequency coupling were not examined. This choice was intentional, as the present study aimed to examine whether different categories of positive affect can be distinguished using physiological indices that are measurable not only in laboratory settings but also in more practical environments. Accordingly, relative power was adopted as a simple and reproducible EEG-based index. Nevertheless, restricting the analysis to relative power inevitably limits the interpretability of the neural mechanisms underlying differences between affective states. Expanding the analytical scope to include additional EEG features represents an important direction for future work. Relatedly, recent studies have demonstrated that deep learning–based approaches can effectively model complex structures in EEG signals for emotion recognition [94,95]. For example, STRFLNet has been proposed to learn spatiotemporal representations from EEG data, particularly in naturalistic and longitudinal settings, while graph-based domain adaptation approaches such as the multi-source selective graph domain adaptation network have focused on improving cross-subject generalization by explicitly modeling inter-subject variability [94,95]. These approaches address methodological challenges that are beyond the scope of the present study. While the present study focused on examining the discriminative capability of simple and interpretable physiological indices under controlled experimental conditions, integrating insights from such advanced modeling approaches may contribute to further developments in physiological research on positive affect.

Fourth, although ICA-based preprocessing is widely adopted as a standard approach for EEG artifact rejection, its effectiveness is generally constrained when applied to low-density EEG recordings. In particular, recent state-of-the-art studies have proposed more advanced and automated methods for EEG artifact identification and correction, including approaches based on machine learning and data-driven classification [96]. While these state-of-the-art methods represent important methodological advances and were considered, they were not incorporated in this study. Future work should systematically compare ICA-based preprocessing with more recent artifact correction approaches and examine their impact on EEG-derived indices, especially in low-channel EEG settings.

Finally, in the experiments conducted in this study, positive affective states were labeled based on self-report, followed by analyses using physiological indicators. Accordingly, data were collected from healthy adults who were considered capable of reporting their own affective states as experimental participants. Because physiological indices are known to vary depending on age and the presence of disease, the findings of this study cannot be considered sufficient to suggest applicability to populations such as older adults or individuals with diseases. Future research should therefore target populations for whom the application of PPIs is expected, including older adults and individuals with diseases.

## 6. Conclusions

This study focused on the distinction between HAPA and LAPA within positive affect. Using a video-based emotion elicitation task, LAPA and HAPA states were compared. Although no significant differences were found in the HRV indices, statistical differences were observed in the EEG indices. Specifically, under the LAPA condition, relative power increased in the theta band in occipital regions, decreased in the alpha band across occipital–parietal regions, and decreased in the beta band at parietal sites. These findings suggest that EEG relative power indices capture systematic group-level differences associated with LAPA. By showing that differences in arousal levels within positive affect can be identified even using relative power, a simple and reproducible index.

Future research should examine the correspondence with stimulus modalities and dimensional models of emotion more rigorously and conduct integrated analyses of diverse EEG indices, including temporal synchrony and cross-frequency coupling. In addition, replication studies with different age groups or clinical populations are needed to evaluate the generalizability of the physiological characteristics of HAPA and LAPA. Such developments are expected to refine frameworks for emotion evaluation that do not depend on subjective reports and contribute to advances in emotional understanding and affective intervention technologies.

## Figures and Tables

**Table 1:** HRV indices used in this study [45].

Indices	Description
SDNN	Standard deviation of NN intervals
RMSSD	Root mean square of consecutive RR interval differences
pNN50	Percentage of successive RR intervals that differ by more than 50 ms

**Table 2:** EEG indices used in this study.

Indices	Frequency Band (Hz)
Theta	4–7 Hz
Alpha	8–12 Hz
Beta	13–30 Hz

**Table 3:** Demographic characteristics and the number of participants.

Generation	Male	Female
20 s	9	9
30 s	9	9 (1)
40 s	9 (1)	9 (2)

Numbers in parentheses indicate the number of excluded participants.

**Table 4:** Effect sizes (*r*) for all Wilcoxon signed-rank test pairs among the eight stimuli.

VideoID	1	2	3	4	5	6	7	8
1	—	0.17	0.49	0.29	0.86	0.87	0.87	0.84
2		—	0.46	0.54	0.85	0.86	0.87	0.82
3			—	0.74	0.86	0.87	0.87	0.86
4				—	0.83	0.87	0.86	0.85
5					—	0.72	0.77	0.47
6						—	0.42	0.21
7							—	0.50
8								—

**Table 5:** Results of the Wilcoxon signed-rank tests for the HRV indices (*p*-values adjusted using the Holm method).

Indices	Mean Different	Mean Difference (95% CI)		*p* Value	Effect Size
		Low	High		
SDNN	1.797	−1.120	5.116	0.263	0.07
RMSSD	0.479	−3.014	4.724	1.000	0.24
pNN50	0.364	−0.562	1.365	1.000	0.09

**Table 6:** The result of ART ANOVA for the EEG frequency bands (*p*-values adjusted using the Holm method).

Indices	Main Effect: Condition	Main Effect: Channel	Interaction
Theta	*p* < 0.001	0.004	0.492
Alpha	*p* < 0.001	*p* < 0.001	0.010
Beta	0.002	*p* < 0.001	0.185

**Table 7:** Wilcoxon test results for each EEG band and channel (*p*-values adjusted using the Holm method).

Indices	Channel	Mean Difference	Mean Difference (95% CI)		*p* Value	Effect Size
			Low	High		
Theta	O1	0.083	0.042	0.125	0.022	0.474
	O2	0.089	0.033	0.140	0.049	0.444
Alpha	O1	−0.125	−0.175	−0.074	0.002	0.545
	O2	−0.159	−0.208	−0.107	*p* < 0.001	0.652
	P3	−0.103	−0.142	−0.063	*p* < 0.001	0.595
	P4	−0.131	−0.171	−0.091	*p* < 0.001	0.687
	Pz	−0.139	−0.180	−0.099	*p* < 0.001	0.700
Beta	Pz	−0.059	−0.093	−0.026	0.030	0.463

## Data Availability

The dataset introduced in this article is part of an ongoing study and is not immediately available. Requests for access to the dataset may be directed to “toshi@shiseido.com”.

## Appendix A. Effect Size and CI for Manipulation Check

Detailed statistical results for analyses with a large number of comparisons are provided in this appendix for clarity and completeness.

**Table A1:** Pairwise post hoc comparisons with effect sizes (*r*) and 95% confidence intervals.

Video-ID Pair		n_Pairs	Mean Difference	Mean Difference (95% CI)		*p* Value	Effect Size
				Low	High		
1	2	50	0.84	−0.260	2.000	0.428	0.165
1	3	50	2.12	1.040	3.240	0.008	0.488
1	4	50	−1.32	−2.700	0.060	0.174	0.288
1	5	50	−7.16	−8.641	−5.740	0.000	0.858
1	6	50	−9.4	−11.000	−7.860	0.000	0.870
1	7	50	−10	−11.600	−8.460	0.000	0.868
1	8	50	−8.64	−10.400	−6.880	0.000	0.837
2	3	50	1.28	0.380	2.220	0.017	0.461
2	4	50	−2.16	−3.260	−1.020	0.002	0.540
2	5	50	−8	−9.580	−6.480	0.000	0.848
2	6	50	−10.24	−11.900	−8.560	0.000	0.855
2	7	50	−10.84	−12.520	−9.180	0.000	0.870
2	8	50	−9.48	−11.440	−7.460	0.000	0.822
3	4	50	−3.44	−4.600	−2.380	0.000	0.735
3	5	50	−9.28	−10.961	−7.600	0.000	0.857
3	6	50	−11.52	−13.300	−9.740	0.000	0.865
3	7	50	−12.12	−13.920	−10.340	0.000	0.865
3	8	50	−10.76	−12.760	−8.780	0.000	0.864
4	5	50	−5.84	−7.280	−4.480	0.000	0.834
4	6	50	−8.08	−9.560	−6.620	0.000	0.867
4	7	50	−8.68	−10.200	−7.180	0.000	0.863
4	8	50	−7.32	−9.060	−5.660	0.000	0.851
5	6	50	−2.24	−2.960	−1.500	0.000	0.715
5	7	50	−2.84	−3.620	−2.060	0.000	0.768
5	8	50	−1.48	−2.360	−0.520	0.013	0.474
6	7	50	−0.6	−1.060	−0.140	0.069	0.422
6	8	50	0.76	−0.060	1.640	0.428	0.209
7	8	50	1.36	0.660	2.140	0.013	0.501

Note. Pairwise comparisons were conducted using Wilcoxon signed-rank tests with Holm adjustment for multiple comparisons. Effect sizes are reported as *r*.
